# Evaluating the Death and Recovery of Lateral Line Hair Cells Following Repeated Neomycin Treatments

**DOI:** 10.3390/life11111180

**Published:** 2021-11-04

**Authors:** Alexandra Venuto, Timothy Erickson

**Affiliations:** 1 Department of Biology, East Carolina University, Greenville, NC 27858, USA; venutoa18@students.ecu.edu; 2 Department of Biology, University of New Brunswick, Fredericton, NB E3B 5A3, Canada

**Keywords:** zebrafish, lateral line, neuromast, hair cell, ototoxicity, toxicity, regeneration, cell death, neomycin, aminoglycosides

## Abstract

Acute chemical ablation of lateral line hair cells is an important tool to understand lateral line-mediated behaviors in free-swimming fish larvae and adults. However, lateral line-mediated behaviors have not been described in fish larvae prior to swim bladder inflation, possibly because single doses of ototoxin do not effectively silence lateral line function at early developmental stages. To determine whether ototoxins can disrupt lateral line hair cells during early development, we repeatedly exposed zebrafish larvae to the ototoxin neomycin during a 36 h period from 3 to 4 days post-fertilization (dpf). We use simultaneous transgenic and vital dye labeling of hair cells to compare 6-h and 12-h repeated treatment timelines and neomycin concentrations between 0 and 400 µM in terms of larval survival, hair cell death, regeneration, and functional recovery. Following exposure to neomycin, we find that the emergence of newly functional hair cells outpaces cellular regeneration, likely due to the maturation of ototoxin-resistant hair cells that survive treatment. Furthermore, hair cells of 4 dpf larvae exhibit faster recovery compared to 3 dpf larvae. Our data suggest that the rapid functional maturation of ototoxin-resistant hair cells limits the effectiveness of chemical-based methods to disrupt lateral line function. Furthermore, we show that repeated neomycin treatments can continually ablate functional lateral line hair cells between 3 and 4 dpf in larval zebrafish.

## 1. Introduction

Fish and some amphibians possess a unique sensory system called the mechanosensory lateral line, which comprises clusters of mechanically sensitive hair cells distributed all over the body in organs called neuromasts. The lateral line detects hydrodynamic information and contributes to several behaviors such as foraging, predator avoidance, and swimming coordination [1,2]. For example, the lateral line is a critical sensory component for larval and adult fish to orient to water flow (rheotaxis) [3,4,5]. Additionally, aquatic organisms can identify locations of predators and prey by combining the direction and force of the water interacting with the neuromast pattern along the body [6,7].

To investigate these lateral line-mediated behaviors, researchers use a variety of physical and chemical strategies to functionally disrupt the lateral line [8,9]. The use of ototoxins to chemically ablate hair cells is the most popular technique due to their specificity, convenience, and applicability to a wide range of aquatic vertebrates. Common ototoxins include aminoglycoside antibiotics (e.g., neomycin), certain heavy metals (e.g., copper), and various platinum derivatives (e.g., cisplatin) [10,11,12,13]. These compounds disrupt the lateral line by entering hair cells through functional mechanotransduction channels, causing oxidative stress, and triggering apoptotic cell death [14,15,16,17,18]. While useful for lateral line research, aminoglycosides are also major causes of irreversible hearing loss in humans [19]. Understanding how zebrafish hair cells respond to ototoxic assaults may lead to preventative or therapeutic strategies to combat human hearing loss [20].

The acute chemical ablation of hair cells is a valuable tool for studies on adult fish and free-swimming larvae. However, there is little information about how the lateral line influences behavior in early-stage larvae, likely because ototoxins are less effective at early developmental stages [21,22]. Previous research suggests two reasons why a single dose of ototoxin is less effective at younger stages compared to older larvae and adults: (i) the rapid proliferation of hair cells at early developmental stages, and (ii) the presence of immature hair cells that are resistant to chemical ablation. Regarding proliferation, initial neuromast deposition in developing zebrafish begins as early as 20 h post-fertilization (hpf), and hair cells start to gain function by 34 hpf [23,24,25,26]. The number of hair cells per neuromast increases from 3 to 5 days post-fertilization (dpf) and results in the generation of over 300 functional hair cells [13,23,26,27]. Therefore, a single ototoxic treatment during this timeframe is unlikely to fully disable the lateral line, especially considering that approximately half of all hair cells are regenerated within 24 h post-treatment [13]. It has been noted that regeneration at this stage of development is difficult to track due to the rapid growth of the lateral line [27]. Regarding ototoxin resistance, susceptibility to chemical ablation requires that hair cells possess functional mechanotransduction channels [28]. Immature hair cells that have not yet developed mechanosensitivity are resistant to the effects of ototoxins [21,22], such as hair cells where mechanotransduction is disabled through chemical or genetic means [29,30]. Developing neuromasts may contain a relatively high proportion of immature hair cells [21,22,31] that can potentially gain mechanosensitivity after ototoxin treatment, allowing neuromasts to partially regain function without regenerating new hair cells. Indeed, following exposure to ototoxin, lateral line-mediated behaviors of zebrafish larvae recover before hair cell regeneration is complete [5,32]. However, whether functional maturation of immature hair cells precedes cellular regeneration has not been tested explicitly.

Recently, we reported the first genetic model for the specific loss of lateral line function in zebrafish [33]. To complement our studies with this mutant, here, we aim to identify a timeline of repeated neomycin treatments in larval zebrafish aged 3–4 dpf that will mimic the congenital loss of lateral line function, while minimizing off-target toxicity. We use two treatment timelines (every 6 or 12 h) with neomycin concentrations from 50 to 400 µM. We compare these treatment regimens in terms of larval survival, efficacy of hair cell ablation, functional maturation of hair cells, and cellular regeneration of hair cells following repeated neomycin treatments. Our data support the hypothesis that rapid functional maturation of ototoxin-resistant hair cells diminishes the effectiveness of ototoxins as a tool to study the lateral line. We also identify a set of treatment conditions that can be used in future studies along with the lateral line mutant to investigate how the lateral line contributes to the behaviors of larval zebrafish prior to 5 dpf.

## 2. Materials and Methods

### 2.1. Ethics Statement and Animal Husbandry

All research involving animals was carried out at East Carolina University (ECU; Greenville, NC, United States) and complied with guidelines stipulated by the ECU Institutional Animal Care and Use Committee. Zebrafish (*Danio rerio*) were maintained and bred using standard procedures [34].

### 2.2. Neomycin Sulfate Treatments

**Figure 1:**
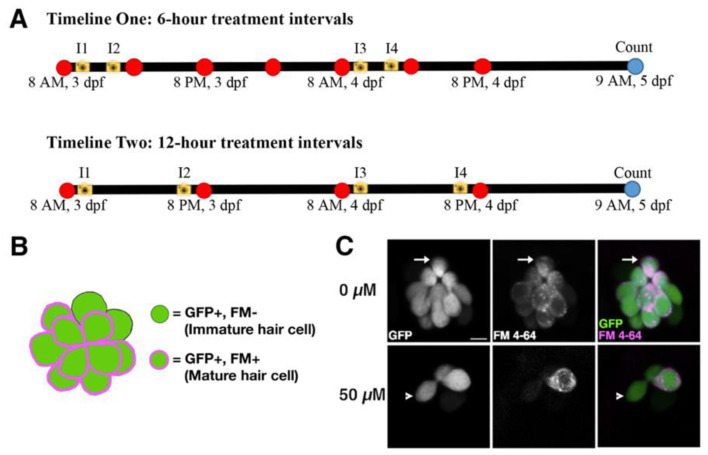
Experimental set-up. (**A**) The 6-h and 12-h treatment timelines with neomycin concentrations of 0, 50, 200, 300, and 400 µM beginning 8 a.m. at 3 days post-fertilization (dpf). Red dots indicate treatment timepoints, yellow cameras indicate imaging timepoints for hair cell counts (I1–I4), and blue dots indicate final count of surviving larvae. (**B**) Representation of a neuromast showing the strategy used to differentiate between mature and immature hair cells. Mature cells are labelled with both GFP and FM 4-64, while immature cells are labelled with GFP, but not FM 4-64. (**C**) Representative confocal images of O2 neuromasts at imaging timepoint I1 in the 0 and 50 µM neomycin treatment groups. The *Tg(myo6b:eGFP-pA)vo68Tg* line is green and FM 4-64 is magenta in the merge of the two channels in the third column. The full arrow indicates a functional hair cell positive for both GFP and FM 4-64. The arrowhead indicates an ototoxin-resistant, immature hair cell that is positive for GFP only. Scale bar = 5 µm. Additional representative images from imaging timepoints I1-I4 are provided in Figure S1.

A 1 mM Neomycin sulfate (EMD Millipore Corp., Burlington, MA, USA) stock solution was made in 25 mL of E3 (embryo medium) and diluted in 35 × 10 mm Petri dishes containing 30 mL E3 to make 50, 100, 200, 300, and 400 µM concentrations for each treatment round. These concentrations are consistent with those previously used as single treatments on larval zebrafish [5,13,22]. Timeline One involved neomycin treatments every six hours for a total of seven treatments, while Timeline Two entailed treatments every twelve hours for a total of four treatments (Figure 1A). Both timelines were repeated three times at all neomycin concentrations. All treatments were carried out in Petri dishes containing 18–25 larvae and incubated for 25 min at 28.5 °C before being returned to Petri dishes with fresh E3.

### 2.3. Survivorship Counts

Starting with the first treatment at 8 a.m. on 3 dpf, the number of surviving larvae in each treatment condition was recorded every 6 h until the final treatment at 8 p.m. on 4 dpf. The final survivorship count was made the following morning at 9 a.m. on 5 dpf. In addition to the final survivorship count on 5 dpf, we quantified incidences of pericardial edema and imaged larvae with a ZEISS SteREO Discovery.V8 Microscope equipped with a Jenoptik Gryphax Arktur camera.

### 2.4. Hair Cell Counts and Strategy to Differentiate between Mature and Immature Hair Cells

Figure 1A outlines the imaging time points for both neomycin treatment timelines. In all cases, the first round of imaging was carried out after an hour of recovery time following the first neomycin treatment (I1). The second round of imaging was carried out an hour before the second neomycin treatment (I2). The third round of imaging was carried out after an hour of recovery time, following the neomycin treatment on the morning of 4 dpf (I3). The fourth and final round of imaging was carried out an hour before the next neomycin treatment on the afternoon/evening of 4 dpf (I4). Hair cell counts were carried out at the 0, 50, 100 and 200 µM concentrations for Timeline One (6-h intervals) and at all concentrations for Timeline Two (12-hout intervals). Timeline One counts did not include the 300 and 400 µM concentrations due to high larval mortality at later treatments. To quantify hair cell numbers, evaluate the efficacy of neomycin treatments, and quantify the functional and cellular regeneration of the lateral line following repeated neomycin treatments, we utilized simultaneous transgenic and vital dye labeling of lateral line hair cells to distinguish between mature, mechanically sensitive hair cells and immature hair cells that had yet to develop a functional transduction apparatus (Figure 1B,C). We mark both immature and mature hair cells using the *Tg(myo6b:eGFP-pA)vo68Tg* line [33], which uses the *myo6b* promoter to drive GFP in all hair cells, beginning at an early stage of their development [35]. Simultaneously, functional hair cells were co-labeled with 3 µM FM 4-64 (Thermo Fisher Scientific) in E3 embryo media for 30 s, followed by rinses in E3. FM 4-64 is far-red fluorescent dye that enters hair cells through functional mechanotransduction channels [30,36]. In this study, we define mature hair cells as being positive for both GFP and FM 4-64 and immature hair cells as being GFP positive but FM 4-64 negative (Figure 1B,C). For all experiments, 5–9 larvae from treated and untreated groups were randomly chosen for imaging. Live larvae were mounted in 1.2% low melting point agarose (IBI Scientific) dissolved in E3. The L1, MI1, and O2 neuromasts were imaged on a Zeiss LSM 800 confocal. After imaging, larvae were freed from the agarose and returned to their respective control or experimental groups. Hair cell counts were carried out using the *z*-stack data.

### 2.5. Statistics

*p*-values of less than 0.05 were considered significant. Analyses were done in RStudio using the R stats and betareg packages [37,38], and plots were made with ggplot2 [39].

## 3. Results

### 3.1. Toxicity of Repeated Neomycin Treatments in 3–4 dpf Zebrafish

**Figure 2:**
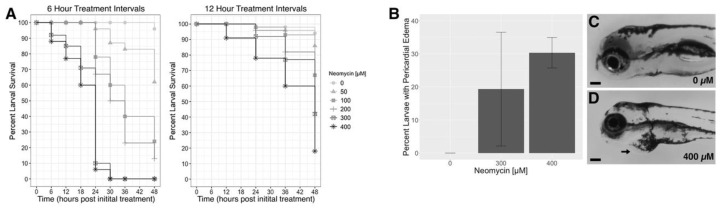
Toxicity of repeated neomycin treatments in zebrafish larvae. (**A**) Percent survival for Timeline One (6-h treatment intervals) and Timeline Two (12-h intervals). Final counts were carried out at 5 dpf, 48 h following the first treatment. (**B**) Average percent of larvae with pericardial edema at 5 days post-fertilization (dpf) after four neomycin treatments given at 12-h intervals. Error bars represent standard deviation. (**C**) Image of an untreated (0 µM neomycin) larva at 5 dpf. (**D**) Image of a 5 dpf larva exhibiting pericardial edema (arrow) after 4 treatments with 400 µM neomycin delivered every 12 h. Scale bar = 0.1 mm.

In the following experiments, we use repeated neomycin treatments delivered at either 6-h or 12-h intervals over the course of 36 h in zebrafish at 3–4 dpf and at neomycin concentrations of 0, 50, 100, 200, 300, and 400 µM (Figure 1A). To assess general toxicity that may be associated with repeated neomycin treatments, we quantified survivorship across treatment schedules and concentrations. Neomycin concentrations of 100 µM or higher result in greater than 50% larval mortality when administered every 6 h (Figure 2A and Table S1). The two highest concentrations, 300 and 400 µM, result in 100% lethality, and only the 50 µM concentration allows for greater than 50% survival using the 6-h treatment timeline. Twelve-hour treatment intervals result in improved survival relative to the 6-h intervals. While only 18% of larvae survive when exposed to 400 µM neomycin every 12 h (*n* = 64), survival was almost 50% at 200 and 300 µM, 67% at the 100 µM, and 86% at 50 µM (Figure 2A and Table S1). Despite increased survival using the 12-h interval, many larvae from the 300 µM and 400 µM neomycin treatment groups exhibited pericardial edema and were smaller in size compared to untreated larvae at 5 dpf (*n* = 6/30 and 5/16 in the 300 and 400 µm groups, respectively) (Figure 2B–D). To determine which factors were culpable for the mortality rates, we ran a generalized linear model regressing percent survival after 24 h against a concentration of neomycin and the experimental timeline used (Table 1). Treatment concentrations 100 µM and greater are significantly associated with increased mortality (*p* < 0.0001), while less frequent treatments favor survival (*p* < 0.0001). Overall, we find that repeated neomycin treatments at 3–4 dpf at concentrations over 50 µM result in high levels of larval mortality.

### 3.2. Hair Cell Proliferation and Maturation in Untreated Larvae

**Figure 3:**
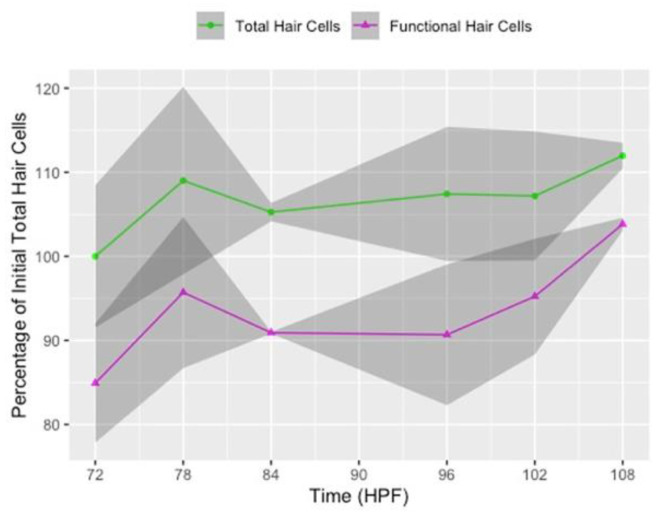
Hair cell proliferation and maturation in untreated (0 µM neomycin) larvae between 3 and 4 days post-fertilization (dpf). Line graph of the percentage of total (GFP+, green line) and functional (FM 4-64+, magenta line) hair cells in untreated larvae relative to the total number of GFP+ hair cells when larvae were 72 hpf (3 dpf) at the beginning of the experiment. Data points are from each imaging timepoint from all control group replicates of the 6- and 12-h timelines combined. The x-axis represents hours post-fertilization (HPF). The shaded regions surrounding the line represent the 95% confidence intervals. Average counts are provided in the 0 µM neomycin rows of Table S2, and counts from individual neuromasts in the 12-h timeline are provided in Figure S2.

Chemical ablation of lateral line function may be less effective in larvae younger than 5 dpf due to the natural proliferation of hair cells at this developmental stage. To determine if neuromasts are still adding new hair cells between 3 and 4 dpf, we quantified the total number of hair cells (GFP+) and the number of functional hair cells (FM+) per neuromast in untreated larvae and expressed these numbers as a percent of the total number of GFP+ hair cells from the beginning of the experiment. There is continuous cell proliferation and functional maturation of lateral line hair cells during the 36-h period between imaging timepoints I1 and I4 in untreated larvae (Figure 3 and 0 µm neomycin rows in Table S2). On average, neuromasts add 1.6 GFP-positive hair cells between imaging timepoints 1 and 4, representing a 12.0% increase during these 36 h of development. Over the same period, neuromasts add an average of two FM-positive cells, a 18.9% increase (Figure 3 and Table S2). The number of functional, FM-positive hair cells is consistently less than the GFP-positive hair cells at all imaging timepoints, yet there is an overall increase in the proportion of FM-positive cells relative to the total number of hair cells per neuromast between imaging timepoints I1 and I4 (FM/GFP = 0.849 for I1, 0.995 for I4; two-tailed *z*-score test for two proportions, *z* = −2.31, *p* = 0.021) (Figure 3). This indicates that a greater proportion of hair cells in each neuromast are mechanically sensitive as development proceeds. Together, these data suggest that, between days 3 and 4 of zebrafish development, the number of hair cells per neuromast has not yet reached steady state, and both nascent and mature hair cells are still being added.

### 3.3. Effect of Neomycin Concentration and Treatment Interval on Hair Cell Death

**Figure 4:**
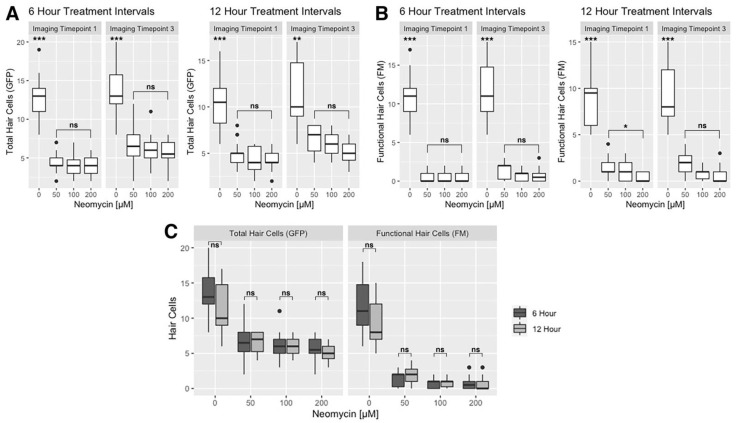
Effect of neomycin concentration and treatment intervals on hair cell death. Boxplots represent the number of hair cells per neuromast for both the 6-h and 12-h treatment timelines. (**A**) Total (GFP+) and (**B**) functional (FM+) hair cells at imaging timepoints I1 and I3. The Kruskal–Wallis ANOVA with Dunn post-test results are provided in Table S3. (**C**) Comparison of the 6-h and 12-h timelines in terms of the number of GFP+ and FM+ hair cells remaining after treatment at imaging timepoint I3 on 4 dpf. There is no significant difference in the hair cell counts between the two treatment timelines (Kruskal-Wallis ANOVA with Dunn post-test, α = 0.05; Table S4). Significance levels are as follows: *** = *p* < 0.001, ** = *p* < 0.01, * = *p* < 0.05, ns = not significant.

To measure the effect of neomycin concentration and treatment interval on the efficacy of hair cell ablation, we quantified the number of GFP-positive and FM-positive hair cells that remain following neomycin exposure at imaging time points I1 and I3. We excluded the 300 and 400 µM neomycin treatments from these analyses due to high mortality and abnormal morphology observed at those concentrations (Figure 2). Consistently, we find that GFP-positive, FM-negative hair cells remain after a 25-min exposure to neomycin at concentrations ranging from 50 to 200 µM (Figure 4A). At imaging timepoints 1 and 3, neomycin concentration did not significantly affect the number of remaining GFP-positive cells using either 6- or 12-h treatment intervals (ANOVA with Dunn post-test, α = 0.05) (Figure 4A, Table S3). Next, looking only at mechanically sensitive (FM-positive) hair cells, neomycin concentration did not have a significant effect on the number of FM-positive cells remaining after treatment at imaging timepoint 1 in either the 6- or 12-h treatment intervals, with the exception of the comparison between the 50 and 200 µM neomycin concentrations (ANOVA with Dunn post-test, *p* = 0.042) (Figure 4B, Table S3). At imaging timepoint 3 within both the 6- and 12-h treatment experiments, neomycin concentration did not have a significant effect on the number of FM-positive cells remaining after treatment (ANOVA with Dunn post-test, α = 0.05) (Figure 4B). In general, we observe a non-significant decrease in the numbers of total and functional hair cells as neomycin concentration increases (Figure 4).

Next, we analyzed whether the frequency of neomycin treatments had an effect on the level of hair cell ablation by comparing the 6- and 12-h timelines in terms of the difference in the number of total and functional hair cells at imaging timepoint 3 for all neomycin concentrations. We find that more frequent treatments do not have a significant effect on cell death (ANOVA with Dunn post-test, α = 0.05) (Figure 4C, Table S4). Overall, we find that mechanically sensitive hair cells in zebrafish at age 3–4 dpf are susceptible to neomycin exposure and can be continuously disrupted over 36 h. Furthermore, neuromasts from 3 to 4 dpf larvae contain immature, neomycin-resistant hair cells that remain after treatment.

### 3.4. Cellular and Functional Regeneration in 3 dpf Larval Zebrafish

**Figure 5:**
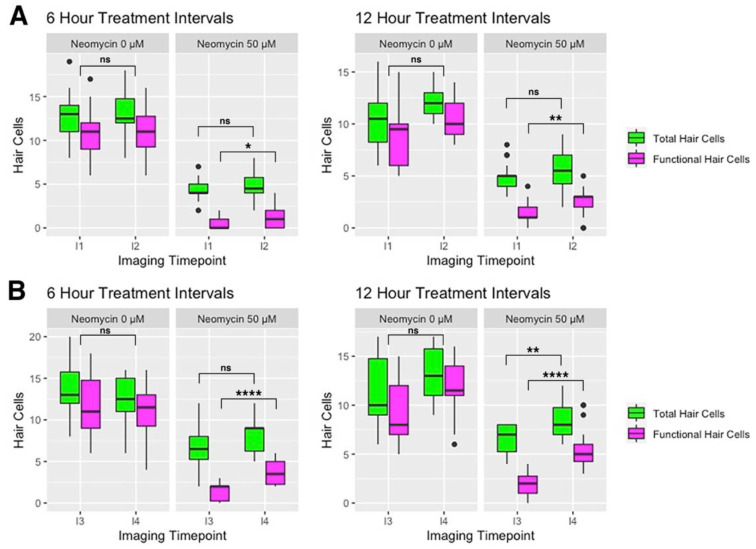
Comparison of hair cell counts in the 0 µM and 50 µM neomycin treatment groups at (**A**) 3 dpf and (**B**) 4 dpf. Green boxplots indicate cell counts using the GFP transgene to mark both transducing and non-transducing hair cells, and the magenta boxplots indicate hair cell counts using FM 4-64 to label mechanically sensitive hair cells. See Figure 1A for timing of imaging timepoints I1-I4. Kruskal–Wallis ANOVA with Dunn post-test was performed to find significance levels, which are as follows: **** = *p* < 0.0001, ** = *p* < 0.01, * = *p* < 0.05, ns = not significant.

We assessed cellular and functional regeneration following exposure to neomycin in 3 dpf larvae by analyzing the data from imaging timepoints I1 and I2. Given the high mortality rates associated with repeated neomycin treatments of 100–400 µM (Figure 2), we focused our analysis on the 50 µM condition. We observe a significant increase in the number of functional (FM-positive) hair cells between time points I1 and I2 for both the 6- and 12-h treatment intervals (Welch’s t-test, 6 h: M = 0.8, SD = 0.4, *p* = 0.028; 12 h: M = 1.0, SD = 0.3, *p* = 0.002) (Figure 5A). There was no significant change in the number of GFP-positive cells between time points I1 and I2 in either the 6- or 12-h experiments (6 h: M = 0.39, SD = 0.71, *p* = 0.407; 12 h: M = 0.56, SD = 0.16, *p* = 0.319) (Figure 5A). We conclude that the surviving immature hair cells start to acquire mechanosensitivity within 6 to 12 h after neomycin treatment and that functional maturation of hair cells outpaces the regeneration of new hair cells at 3 dpf.

### 3.5. Cellular and Functional Regeneration in 4 dpf Larval Zebrafish

Similar to the analyses carried out at 3 dpf, we assessed cellular and functional recovery at 4 dpf using 50 µM neomycin given every 6 or 12 h. Between imaging time points I3 and I4, we observe a significant addition of FM-positive hair cells between 6-h treatments and 12-h treatments at 4 dpf (Welch’s t-test, 6 h: M = 2.2, SD = 0.9, *p* = 0.000016; 12 h: M = 3.9, SD = 0.2, *p* < 0.00001) (Figure 5B). In the 6-h treatment timeline, there is no significant increase in the number of GFP-positive hair cells between imaging timepoints at 4 dpf (M = 1.3, SD = 1.1, *p* = 0.125). However, in the 12-h treatment interval, there is a significant increase in the number of GFP-positive hair cells between I3 and I4 at 4 dpf (M = 1.6, SD = 0.5, *p* = 0.004) (Figure 5B). These data suggest that, after neomycin treatment at 4 dpf, the remaining immature hair cells start to gain functionality within 6 h, while the regeneration of new hair cells begins within 12 h.

### 3.6. Comparison of Hair Cell Death and Recovery between 3 and 4 dpf Zebrafish

**Figure 6:**
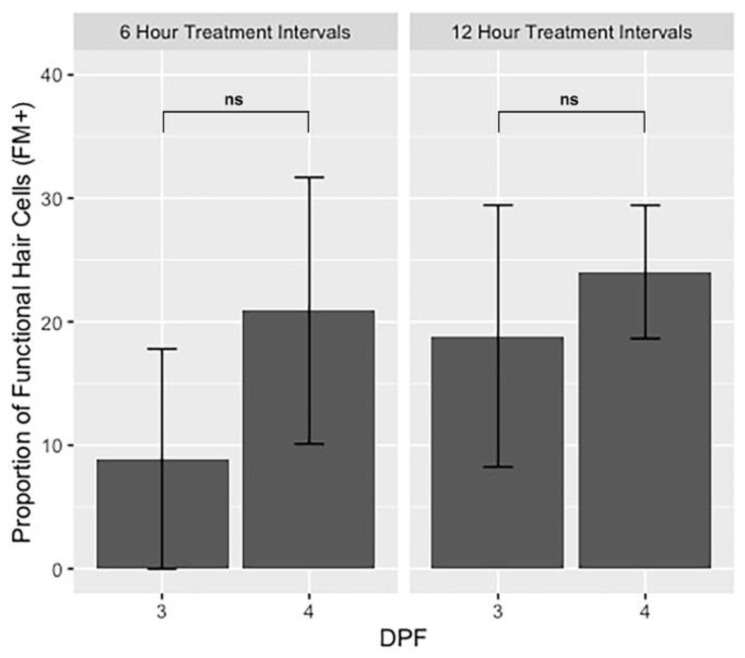
Bar graphs showing the proportion of functional hair cells (FM+) relative to total hair cells (GFP+) one hour after 50 µM neomycin treatments on 3 days post-fertilization (dpf) (I1) and 4 dpf (I3) for both 6-h and 12-h timelines. Error bars represent standard deviation, *n* = 15 for each group. Two-tailed *z*-score test for proportions was used to evaluate the difference between the proportion of hair cells at 3 and 4 dpf. ns = not significant.

Next, we compared the susceptibility of hair cells to 50 µM neomycin at 3 dpf and 4 dpf by quantifying the ratio of functional (FM+) to total (GFP+) hair cells from imaging timepoints I1 (3 dpf) and I3 (4 dpf) for both timelines. Comparing I1 and I3, we observe some variability in the proportions of functional hair cells per neuromast that remain following treatment, but the values are not significantly different in the 6-h or 12-h timelines (two-tailed *z*-score test for two proportions; 6 h: *z* = −0.923, *p* = 0.356; 12 h: *z* = −0.346, *p* = 0.731) (Figure 6). Even though it is not statistically significant, there is a consistent increase in the ratio of FM:GFP from I1 to I3 in both timelines. To determine which factors were responsible for these results, we ran a generalized linear model regressing hair cell proportions against the days post-fertilization and the number of treatments (Table 2). According to this beta-regression model, transitioning from 3 to 4 dpf is associated with an increase in the proportion of mechanically sensitive (FM+) hair cells per neuromast (*p* < 0.001). Conversely, the number of neomycin treatments are significantly associated with a decrease in the proportion of mechanically sensitive cells (*p* < 0.001). Overall, we find that the proportions of functional hair cells that remain after neomycin treatment are statistically similar between 3 and 4 dpf, possibly due to the opposing effects of developmental time and repeated exposures to neomycin.

**Figure 7:**
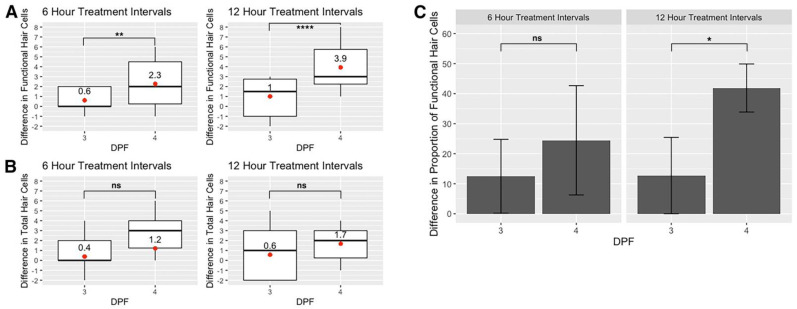
Hair cell recovery at 3 and 4 days post-fertilization (dpf). (**A**) Boxplots showing the difference in functional (FM+) and (**B**) total (GFP+) hair cells between the first and second 50 µM neomycin treatments on 3 dpf (I2 minus I1) and 4 dpf (I4 minus I3) in 6- and 12-h treatment timelines. Red dots and text indicate mean values. Statistical analysis was performed using Kruskal–Wallis ANOVA with Dunn post-test. (**C**) Bar graph showing the difference in the ratio of functional hair cells (FM+) to total hair cells (GFP+) between the first and second treatments on 3 dpf (I2 minus I1) and 4 dpf (I4 minus I3) for 50 µM treatments in 6-h and 12-h treatment timelines. Error bars represent standard deviation, *n* = 15 for each group. One-tailed *z*-score tests for proportions were used to evaluate increases in the proportion of FM+ cells. Significance levels are as follows: **** = *p* < 0.0001, ** = *p* < 0.01, * = *p* < 0.05, ns = not significant.

To assess differences in hair cell proliferation and recovery between 3 and 4 dpf, we compared the change in the number of hair cells between imaging timepoints I1 and I2 (3 dpf) versus the change between I3 and I4 (4 dpf). There is a significant increase in the number of functional, FM+ hair cells added per neuromast after allowing for hair cell recovery between treatments at 4 dpf compared to 3 dpf in both the 6-h and 12-h treatment timelines (Welch’s t-test, 6 h: *p* = 0.007; 12 h: *p* = 0.00009) (Figure 7A). However, there is no significant difference in the total number of GFP+ hair cells per neuromast added between treatments on 4 dpf compared to 3 dpf (6 h: *p* = 0.381; 12 h: *p* = 0.113) (Figure 7B). Looking at the ratio of functional (FM+) to total (GFP+) hair cells per neuromast on days 3 and 4, there is a significant increase in the proportion of functional hair cells after a 12-h recovery on day 4 relative to day 3, but no significant increase after 6-h recovery times (one-tailed *z*-score test for two proportions, 6 h: *z* = −0.845, *p* = 0.199, 12 h: *z* = −1.792, *p* = 0.0363) (Figure 7C). Overall, we find that functional recovery of lateral line hair cells occurs more quickly at 4 dpf compared to 3 dpf in zebrafish larvae.

## 4. Discussion

In this study, we use repeated neomycin treatments to continuously ablate sensory hair cells of the lateral line in 3–4 dpf zebrafish larvae. We find that immature, ototoxin-resistant hair cells remain after neomycin treatment, even after multiple exposures delivered every 6 or 12 h. Our evidence suggests that these immature hair cells gain mechanosensitivity during the inter-treatment intervals, and that functional maturation of nascent hair cells occurs more rapidly at 4 dpf compared to 3 dpf. To our knowledge, this is the first study to analyze hair cell death and recovery using repeated ototoxin treatments on 3–4 dpf zebrafish larvae. Overall, repeated neomycin treatments can be used to continuously disrupt mechanically sensitive hair cells of the lateral line at this early stage of zebrafish development.

### 4.1. Toxicity Associated with Repeated Neomycin Treatments

Single exposures to ototoxins are accepted to be well tolerated by larval and adult fish, thereby allowing for their use in studies of lateral line-mediated behaviors [8]. However, our data clearly demonstrate that repeated neomycin treatments are toxic to larval zebrafish. Survival was less than 50% in both the 100–400 µM neomycin groups treated every 6 h and in the 200–400 µM neomycin groups treated every 12 h. Furthermore, larval death occurred more rapidly with increased number and frequency of treatments (Figure 2). We consistently observe a major decline in larval survival between treatments four and five. This trend was apparent in both experimental timelines, despite differing treatment intervals. In the 300 µM and 400 µM neomycin treatment groups, surviving larvae often exhibit pericardial edema and developmental delays. Incidences of edema were variable between experimental replicates in the 12-h, 300 µM group, suggesting that this condition is near the level where larvae begin to experience this toxic effect of neomycin. Our findings are consistent with other studies that have demonstrated off-target toxicity of compounds commonly used in lateral line research [40,41,42,43,44]. Therefore, we conclude that there is a toxicity threshold for larval zebrafish at 3–4 dpf that is surpassed after four treatments when using neomycin concentrations 100 µM and above.

### 4.2. Susceptibility of Lateral Line Hair Cells to Repeated Neomycin Treatments at 3–4 dpf

Hair cells of the lateral line are rapidly proliferating and maturing at 3–4 dpf [13,26,27]. Mackenzie and Raible (2012) reported an increase in the number of functional hair cells per neuromast between 3 and 4 dpf and that the number of functional hair cells is significantly less than the total number of hair cells per neuromast at 5 dpf. At 3–4 dpf, we similarly find that the total complement of hair cells within a neuromast is always greater than the number of functional hair cells. Between 3 and 4 dpf, neuromasts in untreated larvae exhibit a 12% gain in total cells and 19% increase in functional cells over the same period. These results indicate that, as development proceeds, an increased proportion of hair cells per neuromast are mechanically sensitive and, therefore, potentially susceptible to ototoxins. Furthermore, we find that these mechanically sensitive hair cells are susceptible to neomycin in 3–4 dpf zebrafish and, in agreement with previous studies, that hair cell loss is incomplete due to functionally immature hair cells being resistant to the ototoxic effects of neomycin (Figure 4 and Figure 5) [21,22,27,31].

### 4.3. Hair Cell Maturation and Regeneration after Repeated Neomycin Treatments at 3–4 dpf

Previous studies have provided support for hair cell regeneration occurring more quickly in young larvae than in older larvae or adult fish [27,45,46]. In our study, we observe that there is a significant addition of functional hair cells within 6 h post-treatment at both 3 and 4 dpf (Figure 7). The increase in functional hair cells outpaces the addition of new GFP+ hair cells at both 3 and 4 dpf. This is likely due to the functional maturation of immature GFP+ hair cells remaining after treatment. However, we found that the addition of both functional and total hair cells occurred more quickly at 4 dpf than 3 dpf. Using the 50 µM neomycin treatments, the ratio of functional to total (FM+/GFP+) hair cells increases by approximately 12% in 6 h and 13% in 12 h on 3 dpf. On 4 dpf, the proportion of functional to total (FM+/GFP+) hair cells increases by approximately 25% in 6 h and 42% in 12 h. We saw that in untreated larvae, the number of hair cells per neuromast increases from 3 to 4 dpf. Therefore, the rate of functional and total hair cell addition following neomycin treatment at 4 dpf may be quicker than at 3 dpf due to a natural increased pace of hair cell proliferation. To confirm this, further studies investigating hair cell proliferation at 3 and 4 dpf are necessary.

### 4.4. Mimicking the Chronic Loss of Lateral Line Function in Larval Zebrafish at 3–4 dpf

To our knowledge, only one study has used repeated ototoxic treatments to study the long-term loss of lateral line-mediated behaviors [47]. However, this study was conducted on fish older than 5 dpf, and there may be lateral line-mediated behaviors in younger larvae (prior to swim bladder inflation) that have not been investigated. Given that even partial regeneration can restore lateral line function and behaviors [5,32,46], a single exposure to ototoxin is not an effective way to assess such behaviors in early-stage larvae when hair cells are actively proliferating and maturing. Therefore, repeated treatments appear necessary, but the feasibility of multiple treatments during lateral line formation has not been previously tested.

Our results support using 50 µM neomycin every 12 h to continuously disrupt hair cells of the lateral line in 3–4 dpf zebrafish larvae. Poor survivorship precludes the use of repeated neomycin treatments at concentrations of 100 µM and greater, particularly when delivered every 6 h (Figure 2). Furthermore, neomycin concentrations between 50 and 400 µM are similar in their ability to kill mechanosensitive hair cells (Figure 4), suggesting there is little benefit to using higher concentrations. Although there is a statistically significant addition of functional hair cells between treatments for both the 6-h and 12-h intervals, we observe little to no cellular regeneration (additional GFP+ hair cells) during the same intervals. Considering these results, and because rheotactic behaviors begin to recover approximately 12 h after exposure to neomycin at 5 dpf [32], we advise treating 3–4 dpf larvae with 50 µM neomycin every 12 h to continuously disrupt lateral line hair cells, while minimizing toxic side effects. This treatment regimen may complement the *lhfpl5b* zebrafish mutant in analysis of lateral line-mediated behaviors in early-stage larvae [33].

## Figures and Tables

**Table 1:** Results of a beta regression model regressing percent survival at 24 h against neomycin concentration and treatment timeline to show which variables are significantly contributing to larval survival or mortality (**** = *p* < 0.0001, ns = not significant).

Beta	Estimate	Standard Error	*p* Value	Significance Level
Intercept (0 µM, 6 h)	4.1322996	0.4913248	4.08 × 10^−17^	****
50 µM Neomycin	−0.9457874	0.5789734	0.102	ns
100 µM Neomycin	−2.6188895	0.5660887	3.72 × 10^−6^	****
200 µM Neomycin	−3.2026104	0.5625068	1.24 × 10^−8^	****
300 µM Neomycin	−5.2428102	0.6140916	1.37 × 10^−17^	****
400 µM Neomycin	−5.8515287	0.6317583	2.00 × 10^−20^	****
12-Hour Treatment Intervals	2.5645373	0.3409579	5.41 × 10^−14^	****

**Table 2:** Results of a beta regression model regressing FM:GFP hair cell proportions against the days post-fertilization (dpf), treatment timeline, and number of treatments to show which factors were significant in contributing to the observed proportions of FM+:GFP+ hair cells between 3 and 4 dpf (**** = *p* < 0.0001, ns = not significant).

Beta	Estimate	Standard Error	*p* Value	Significance Level
Intercept (0 µM, I1)	0.1024	0.1330	0.441	ns
4 dpf	1.1435	0.2106	5.68 × 10^−8^	****
Number of Treatments	−0.6593	0.0655	<2 × 10^−16^	****

## Data Availability

The raw data generated in the study are available upon request from the corresponding author.

## Supplementary Materials

The following are available online at https://www.mdpi.com/article/10.3390/life11111180/s1, Figure S1: Representative confocal images of O2 neuromasts at imaging timepoints I1–I4 in the 0 and 50 μM neomycin treatment groups. The *Tg(myo6b:eGFP-pA)vo68Tg* line is green and FM 4-64 is magenta in the merge of the two channels in the third column of each panel. Scale bar = 5 μm for all images. Figure S2: Box plot of total hair cell counts (GFP-positive) from individual neuromasts L1, MI1, and O2 at each imaging timepoint in the 12-h treatment timeline. Table S1: Average final percent larval survival (±standard deviation) at each neomycin concentration. The starting number of larvae are provided for each condition. Table S2: Average number of hair cells (± standard deviation) at imaging timepoints I1–I4 (See Figure 1A) for neomycin concentrations 0, 50, 100, and 200 μM. Table S3: Related to Figure 4A,B—Results of a Kruskal-Wallis ANOVA with Dunn post-test comparing hair cell death between the 50, 100, and 200 μM neomycin concentrations at imaging timepoints I1 and I3 in the 6-h and 12-h treatment timelines. Table S4: Related to Figure 4C—Results of a Kruskal-Wallis ANOVA with Dunn post-test comparing the 6-h treatment timeline to the 12-h treatment timeline at imaging timepoint 3 for both the total (GFP+) and functional (FM+) hair cells at the 50, 100, and 200 μM neomycin concentrations.
