# Supplementary material for: Evaluating the Death and Recovery of Lateral Line Hair Cells Following Repeated Neomycin Treatments
Source: Life (Basel). 2021 Nov 4;11(11):1180. doi: 10.3390/life11111180 (PMC8625531; doi:10.3390/life11111180)
Supplement: Supplementary file 1 [file life-11-01180-s001.zip › Venuto and Erickson Supplementary material Life v2.pdf]

life

MDPI

Venuto and Erickson, Supplementary Materials: ***Figures S1 and S2, Tables S1-S4.***

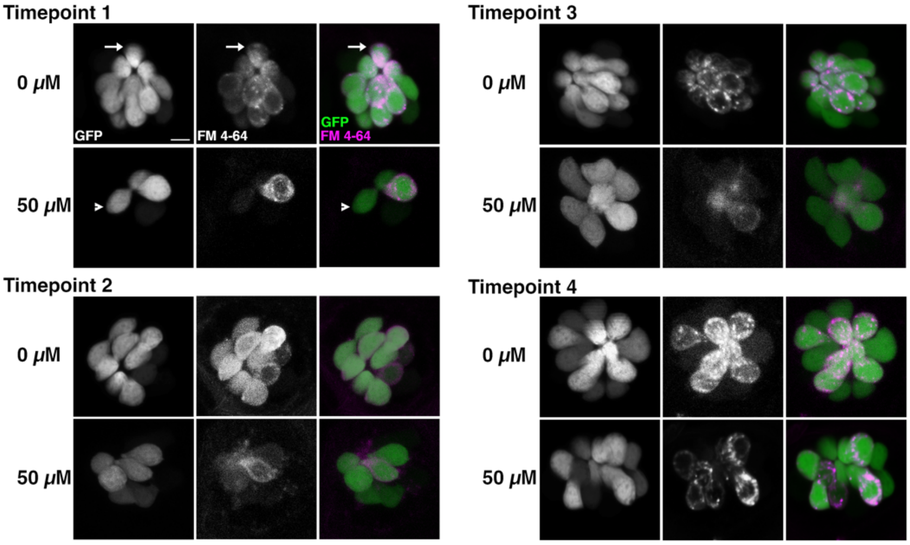

Figure S1. Representative confocal images of O2 neuromasts at imaging timepoints I1-I4 in the 0 and 50 μM neomycin treatment groups. The *Tg(myo6b:eGFP-pA)vo68Tg* line is green and FM 4-64 is magenta in the merge of the two channels in the third column of each panel. Scale bar = 5 μm for all images.

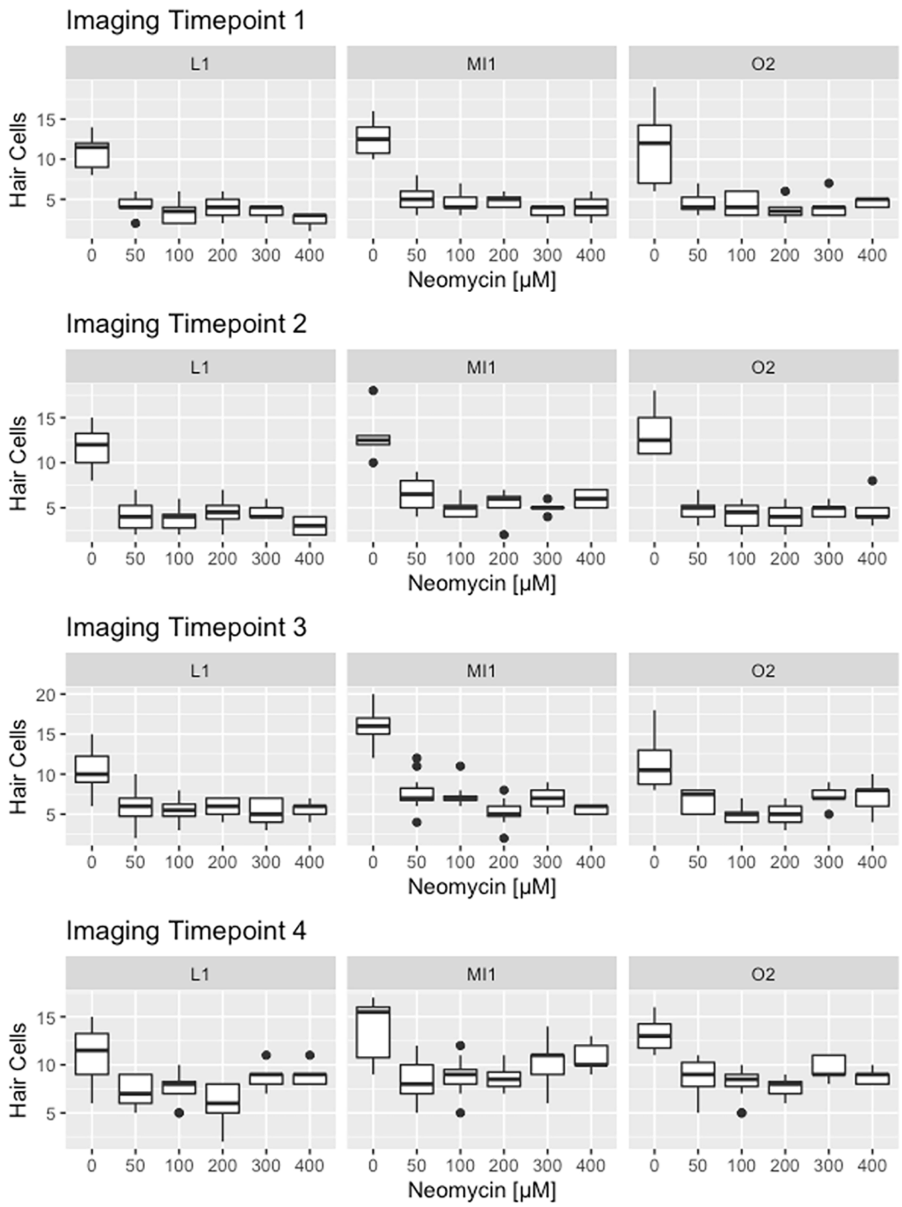

Figure S2. Box plot of total hair cell counts (GFP-positive) from individual neuromasts L1, MI1, and O2 at each imaging timepoint in the 12-hour treatment timeline.

Table S1. Average final percent larval survival (± standard deviation) at each neomycin concentration. The starting number of larvae are provided for each condition.

Neomycin Concentration	6 Hours		12 Hours	
0 µM	96% ± 6.9%	(n = 46)	94% ± 11.0%	(n = 49)
50 µM	62% ± 45.2%	(n = 52)	86% ± 18.7%	(n = 49)
100 µM	24% ± 33.3%	(n = 58)	67% ± 34.4%	(n = 53)
200 µM	13% ± 13.7%	(n = 64)	43% ± 41.8%	(n = 56)
300 µM	0% ± 0.0%	(n = 68)	42% ± 20.2%	(n = 62)
400 µM	0% ± 0.0%	(n = 68)	18% ± 6.7%	(n = 62)

Table S2. Average number of hair cells (± standard deviation) at imaging timepoints I1 – I4 (See Figure 1A) for neomycin concentrations 0, 50, 100, and 200 µM.

	Total Hair Cells (GFP+)	Functional Hair Cells (FM+)	Total Hair Cells (GFP+)	Functional Hair Cells (FM+)
**Timepoint I1**	**6 Hours**	**6 Hours**	**12 Hours**	**12 Hours**
0 µM	12.7 ± 2.7	10.7 ± 2.8	10.6 ± 2.9	9.0 ± 2.8
50 µM	4.3 ± 1.3	0.4 ± 0.4	5.0 ± 1.3	1.5 ± 1.0
100 µM	4.1 ± 1.3	0.4 ± 0.4	4.3 ± 1.4	1.5 ± 1.1
200 µM	3.8 ± 1.3	0.4 ± 0.4	4.3 ± 1.1	0.3 ± 0.3
**Timepoint I2**				
0 µM	12.4 ± 2.7	11.1 ± 2.7	11.4 ± 1.5	10.2 ± 1.9
50 µM	4.7 ± 1.5	1.2 ± 1.2	5.6 ± 1.9	2.5 ± 1.3
100 µM	4.3 ± 1.5	0.8 ± 0.8	4.6 ± 1.2	2.0 ± 1.3
200 µM	4.3 ± 1.6	1.0 ± 1.0	5.1 ± 1.3	2.1 ± 1.2
**Timepoint I3**				
0 µM	12.9 ± 3.1	11.7 ± 3.5	12.2 ± 3.7	10.2 ± 3.3
50 µM	6.8 ± 2.5	1.5 ± 1.2	6.7 ± 1.3	1.7 ± 1.2
100 µM	5.9 ± 1.9	0.8 ± 0.6	5.9 ± 1.4	0.8 ± 0.7
200 µM	5.6 ± 1.5	0.7 ± 0.7	5.2 ± 1.1	0.2 ± 0.2
**Timepoint I4**				
0 µM	13.6 ± 2.9	11.6 ± 3.4	13 ± 2.6	12.1 ± 2.8
50 µM	8.1 ± 2.0	3.7 ± 1.5	8.3 ± 1.9	5.6 ± 2.1
100 µM	8.0 ± 1.8	2.3 ± 1.5	8.4 ± 1.7	4.1 ± 2.1
200 µM	7.2 ± 2.1	1.6 ± 1.1	7.7 ± 1.7	2.8 ± 2.2

Table S3. Related to Figures 4A, B – Results of a Kruskal-Wallis ANOVA with Dunn post-test comparing hair cell death between the 50, 100, and 200 μM neomycin concentrations at imaging timepoints I1 and I3 in the 6-hour and 12-hour treatment timelines.

Total Hair Cells (GFP+) at I1

Conc. 1	Conc. 2	Interval	P-value
50 μM	100 μM	6 Hours	1
50 μM	200 μM	6 Hours	1
100 μM	200 μM	6 Hours	1
50 μM	100 μM	12 Hours	1
50 μM	200 μM	12 Hours	1
100 μM	200 μM	12 Hours	1

Total Hair Cells (GFP+) at I3

Conc. 1	Conc. 2	Interval	P-value
50 μM	100 μM	6 Hours	1
50 μM	200 μM	6 Hours	1
100 μM	200 μM	6 Hours	1
50 μM	100 μM	12 Hours	1
50 μM	200 μM	12 Hours	0.107
100 μM	200 μM	12 Hours	1

Functional Hair Cells (FM+) at I1

Conc. 1	Conc. 2	Interval	P-value
50 μM	100 μM	6 Hours	1
50 μM	200 μM	6 Hours	1
100 μM	200 μM	6 Hours	1
50 μM	100 μM	12 Hours	1
50 μM	200 μM	12 Hours	0.042
100 μM	200 μM	12 Hours	0.077

Functional Hair Cells (FM+) at I3

Conc. 1	Conc. 2	Interval	P-value
50 μM	100 μM	6 Hours	1
50 μM	200 μM	6 Hours	0.462
100 μM	200 μM	6 Hours	1
50 μM	100 μM	12 Hours	1
50 μM	200 μM	12 Hours	0.197
100 μM	200 μM	12 Hours	1

Table S4. Related to Figure 4C – Results of a Kruskal-Wallis ANOVA with Dunn post-test comparing the 6-hour treatment timeline to the 12-hour treatment timeline at imaging timepoint 3 for both the total (GFP+) and functional (FM+) hair cells at the 50, 100, and 200 μM neomycin concentrations.

Neomycin Concentration	Hair Cell Status	P-value
50 μM	GFP +	0.898
100 μM	GFP +	0.884
200 μM	GFP +	0.321
50 μM	FM+	0.719
100 μM	FM+	0.485
200 μM	FM+	0.778
